# A rationale for surgical debulking to improve anti-PD1 therapy outcome in non small cell lung cancer

**DOI:** 10.1038/s41598-019-52913-z

**Published:** 2019-11-15

**Authors:** Florian Guisier, Stephanie Cousse, Mathilde Jeanvoine, Luc Thiberville, Mathieu Salaun

**Affiliations:** 1 Service de pneumologie, oncologie thoracique et soins intensifs respiratoires, CHU Charles Nicolle, Rouen, France; 2 LITIS QuantIF EA4108, Normadie Univ, Rouen, France; 3 INSERM CIC 1404, CHU Charles Nicolle, Rouen, France

**Keywords:** Non-small-cell lung cancer, Surgical oncology

## Abstract

Anti-PD1 immunotherapy has emerged as a gold-standard treatment for first- or second-line treatment of stage IV NSCLC, with response rates ranging from 10 to 60%. Strategies to improve the disease control rate are needed. Several reports suggested that debulking surgery enhances anti-tumor immunity. We aimed at examining tumor burden as a predictive factor of anti-PD1 tretment efficacy and to evaluate the role of cytoreductive surgery in anti-PD1 treated NSCLC. Immunocompetent DBA/2 mice engrafted with various amount of allogeneic lung squamous cancer KLN-205 cells were treated with anti-PD1 monoclonal antibody. Mice engrafted with two tumors also underwent a debulking surgery or a sham procedure. Tumor volume was monitored to assess treatment efficacy. Tumor infiltrating lymphocytes were assessed by flow cytometry. In a retrospective study of 48 stage IV NSCLC patients treated with Nivolumab who underwent a 18-FDG PETscan before treatment onset, the prognostic role of metabolic tumor volume was analysed. Anti-PD1 treatment effect was greater in mice bearing smaller tumors. Treatment with higher doses of anti-PD1 antibody did not improve the outcome, independently of the size of the tumor. In mice bearing 2 tumors, excision of 1 tumor improved the anti-PD1 treatment effect on the remaining tumor. In 48 NSCLC patients receiving anti-PD1 treatment, high metabolic tumor volume was associated with poor overall survival and the absence of clinical benefit. Treg infiltration, but not effector T cells, was positively correlated to tumor volume. Taken together, our results suggest that tumor volume is a predictive factor of anti-PD1 efficacy in NSCLC. Additionally, an experimental murine model suggests that tumor debulking may improve control of residual tumor.

## Introduction

Anti-PD1 immunotherapy has emerged as a gold-standard treatment for first- or second-line treatment of stage IV NSCLC, either in monotherapy or in combination with chemotherapy. However, the majority of patients experiences primary resistance. Strategies to improve the disease control rate are needed. Among them, combination with other immunotherapeutics agents, chemotherapies and/or anti-angiogenic drugs have been reported. The combination of radiotherapy with anti-PD1 treatment is also under investigation, based on a strong biological rationale and case reports of abscopal effect.

Cytoreductive “debulking” surgery has long been considered a standard of care for eligible patients with metastatic renal cell carcinoma and is still widely recommended for selected patients^1,2^. This recommandation relies on many retrospective data and on two prospective clinical trials showing an improved overall survival in patients undergoing nephrectomy before receiving Interferon therapy^3,4^. The mechanisms by which nephrectomy may improve survival in this setting are unclear^5^. Several reports suggested that debulking surgery enhances anti-tumor immunity: (i) reports of spontaneous metastasis regression in patients who underwent primary tumor removal^6–8^, (ii) animal data showing lower immune cytotoxicity and reduced B-cell funciton when the primary tumor is not removed^9,10^ and (iii) studies showing enhanced cell-mediated immunity in patients after nephrectomy^11–13^.

More recently, Oronsky *et al*. reported three cases of abscopal effect after surgery in patients that were previously treated with an anti-PD1 agent^14^. In all three cases, tumor was progressing at the time of surgery. For two of them, anti-PD1 was continued after surgery. In this setting, surgery may contribute to the enhancement of antitumor immunity by anti-PD1 agents by (i) the releasing tumor antigens into the circulation, (ii) the induction of cytokine and chemokine secretion, (iii) the removal of a large amount of immunosuppressive cells and (iv) the more effective mobilisation of the pool of immune cells to a smaller tumoral target.

The aims of our study were to determine wether the tumor burden is a predictive factor of anti-PD1 monoclonal antibody efficacy and to evaluate the role of cytoreductive surgery in anti-PD1 treated NSCLC.

## Methods

### Cell line

KLN-205 cell line (derived from lung squamous cell carcinoma in DBA/2 mouse strain, ref ECACC 90110519) was purchased from Sigma Aldrich (Gillingham, UK). Cells were cultured in Dubelco’s Modified Eagle Medium (Sigma Aldrich) supplemented with 10% serum and 2 mM Glutamine.

### Tumor allografts

The study protocol was approved by the ethics committee for animal research (Comité d’ethique Normandie en matière d’expérimentation animale, C2Ea-54, agreement #2016-110316032111). All experiments were performed in accordance with relevant guidelines and regulations, namely the European Directive 2010/63/EU. Mice were bred in the animal facility of our institution (national agreement #C76-540-05), with unlimited access to food and water, and light/darkness cycle of 12 h/12 h.

For each tumor allograft, cells were collected by trypsinisation and washed twice in PBS. 1.10^4^ to 1.10^7^ cells were resuspended in 100 μL PBS and subcutaneously injected in the flank of a 6 to 12-week-old female DBA/2 mouse (Charles River, L’Abresles, France).

Tumor engraftment led to visible tumors at 1 week in 95% of mice. Tumors were measured every 2–3 days using a caliper by researchers blinded for treatment allocation. Tumor volume was calculated as [length*(width)^2^]/2.

At day 35 after tumor allograft, mice were euthanized and tumors were harvested for further analysis. Euthanasia was performed immediately after the last procedure by mean of 0.8 g/kg Phenobarbital intra-peritoneal injection.

### Treatment regimens

Treatment allocation group was random. Random allocation sequence was generated using a random numbers table and the animal ID numbers. Six animals were used in each group (i.e. mice receiving the same number of cells and the same treatment). Mice received either anti-PD1 (J43 clone, BioXCell, West Lebanon, New Hampshire, USA) or isotype IgG (BioXCell), 100 µg every 7 days or 200 µg twice a week, starting on day 14 after tumor allograft. Treatment administration was not blinded but tumor measurement was.

In the debulking experiments, mice underwent procedure under anesthesia at day 14. A preventive intraperitoneal injection of 0.1 mg/kg Buprenorphine was given before the procedure. Animal anesthesia was performed with inhaled isoflurane. The right flank area was shaved and cleaned with 0.2% chlorhexidine. A 0.5 to 1 cm skin incision was performed ahead of the right flank tumor. Excision of the tumor was performed in half of the mice. The incision was closed with Vetbond glue (3 M, Maplewood, Minnesota, USA) in all mice. Mice were monitored daily and provided with repeated Buprenorphine injections if necessary.

The endpoint was the relative tumor volume at day 21 after treatment initiation. Relative tumor volume was calculated by the following ratio: [volume of an individual tumor at the given time point]/[volume at the time of treatment initiation]. For comparisons in the debulking experiments, only left tumors were taken into account.

### Flow cytometry

KLN-205 tumors from DBA/2 allografted mice (n = 9) were harvested at day 21 after treatment initiation and measured using a caliper to determine tumor volume. Single cell suspension of the harvested tumor was prepared with Gentle MACS dissociator (Miltenyi Biotec, Bergisch Gladbach, Germany) and the corresponding Tumor Dissociation Kit (Miltenyi Biotec), according to manufacturer’s instructions. Cell suspension was passed through a 70- μm cell strainer. Cells (5.10^5^) were incubated with 5 μl/ml purified rat anti-mouse CD16-CD32 mAb (TruStain fcX, Biolegend Inc, San Diego, CA) before incubation with specific anti-mouse antibodies. Cells were marked with CD3-FITC, CD4-APC-Cy7, and CD25-PE (Biolegend Inc), CD8-PerCP-Cy5.5 and FoxP3-APC (eBioscience Affymetryx, Santa Clara, CA, USA), washed with PBS and fixed with 0.5% paraformaldehyde. Flow cytometry analysis was performed on FACS LSR Fortessa (BD biosciences, San Jose, CA, USA), and using FlowJo software (Tree Star, Ashland, OR). Cells were gated, based on forward and side scatter and on living cells. Acquisition of multiparameter data was carried out with an appropriate forward scatter (FSC) threshold to exclude debris. At least 50,000 CD3+ cells per sample were analyzed. Gating strategy is illustrated in Supplementary Fig. S1. T regulatory cells were defined as Foxp3/CD4/CD3 positive cells of all living cells.

### Clinical study

All patients who were treated with Nivolumab as second- or third-line treatment for stage IV NSCLC at the Rouen University Hospital between February 2015 and July 2017 were screened. Patients who underwent 18-FDG PET-scan within the 3 months prior to Nivolumab treatment onset were selected for further analysis. Clinical data, imaging, pathology results and molecular analysis were collected from the electronic medical record. 18-FDG PET-scan were reviewed and Metabolic tumor volume was determined using PET VCAR semi-automatic software of AW server 3.2 (General Electric®, Milwaukee, USA) for segmentation. Non malignant FDG avidity areas were not included in this analysis. MTV cut-off to segregate low *versus* high MTV patients was determined using ROC curve analysis for overall survival.

The protocol received approval from our Institutional Review Board (CHB review board for non-interventional research, agreement #1809B). All research was performed in accordance with relevant guidelines/regulations, namely the European Directive 2014/536/EU and the French law 2012-300 regulating biomedical research.

### Statistical analysis

Statistical analysis was performed using the R language and environment for statistical computing (version 3.1.3, R foundation for statistical computing, Vienna, Austria) on RStudio software (version 0.98.1103, RStudio®, Boston, MA, USA). Data were summarized using mean and standard error for mean.

In the animal experiments, giving the limited sample size and the high number of groups, we used the non-parametric Kruskall and Wallis test (non-parametric equivalent of the one-way analysis of variance - ANOVA-) for multiple group comparison of the relative tumor volume at day 21 post-treatment initiation. Results were considered statistically significant when p < 0.05.

In the clinical dataset, Kaplan-Meier method was used to estimate progression-free survival (PFS). MTV, age, histology and Performance Status were compiled in a proportional hazard Cox model to perform multivariate analysis. Comparisons of the 6-months PFS and 1-year OS between groups were performed using Student t-test. Alpha risk was set to 0.05. In accordance to Bonferonni correction for the performance of 3 comparisons, results were considered statistically significant when p < 0.0167. Clinical benefit was defined as PFS > 6 months.

### Ethics approval

The protocol received approval from our Institutional Review Board (CHB review board for non-interventional research, agreement #1809B). All research was performed in accordance with relevant guidelines/regulations, namely the European Directive 2014/536/EU and the French law 2012-300 regulating biomedical research. Under the French law 2012-300 at the time of data collection, patients’ consent is not required for retrospective analysis of routinely acquired data.

## Results

**Figure 1:**
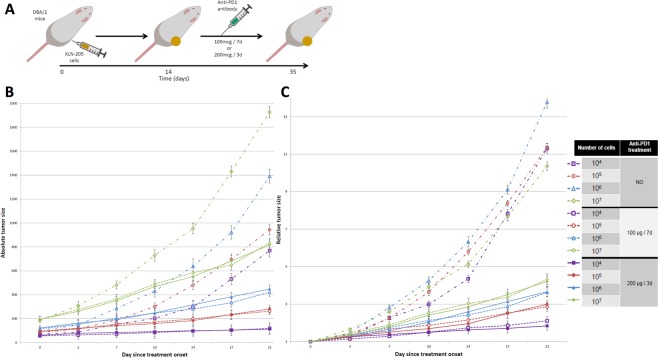
Anti-PD1-induced delay in tumor growth is enhanced in small tumors, independently of treatment schedule. (**A**) Experimental design. DBA/2 mice bearing allogenic KLN-205 squamous lung cancer tumor allografts originated from injection of 10^4^ to 10^7^ cells were treated with intra-peritoneal anti-PD1 (100 µg per week or 200 µg twice a week from day 14 after allograft). Tumor size was measured twice a week with caliper. Control mice were treated with isotype control. Six animals were used in each group (i.e. mice receiving the same number of cells and the same treatment). Error bars represent the standard error for mean. (**B**) Absolute tumor volume during treatment with anti-PD1. (**C**) Tumor volume relatively to the volume at treatment initiation. (**D**) Legend for (**B**,**C**) graphes.

To investigate the influence of tumor burden on the efficacy of anti-PD1 agent, we injected 1.10^4^ to 1.10^7^ KLN-205 cells in allogeneic DBA/2 mice that we treated with intra-peritoneal injection of anti-PD1 monoclonal antibody. In all groups, the anti-PD1 treatment resulted in slower tumor growth (Fig. 1). In mice receiving fewer tumor cells, the treatment effect was greater than in mice receiving 1.10^7^ cells (relative tumor volume at day 21 after treatment initiation: 1.97 vs 4.26, p < 0.05). Treatment with higher doses of anti-PD1 antibody did not improve the outcome, independently of the number of cells engrafted (Fig. 1).

**Figure 2:**
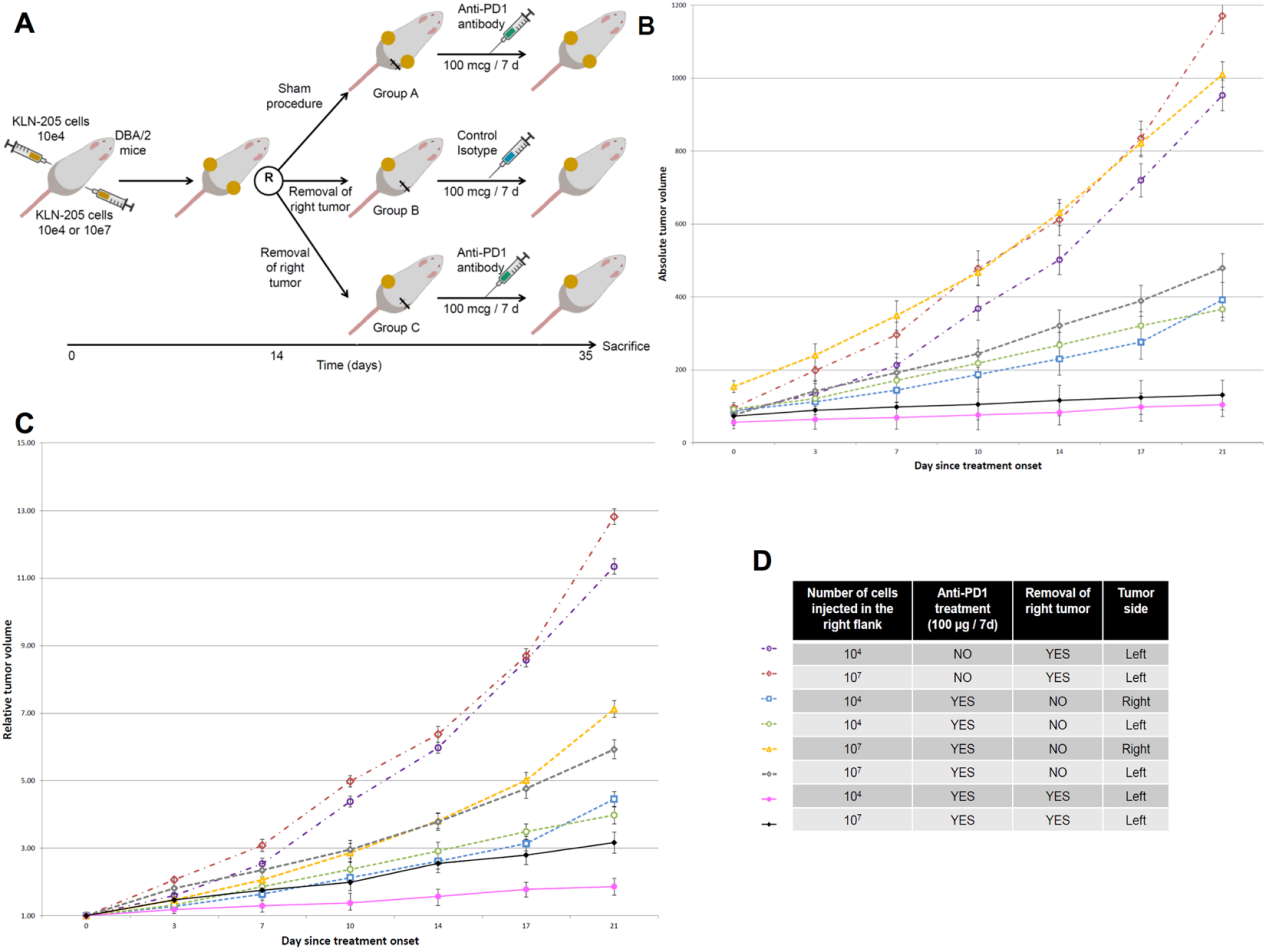
Cytoreductive surgery enhances anti-PD1 treatment effect. (**A**) Experimental design. DBA/2 mice bearing allogenic KLN-205 squamous lung cancer tumor allografts originated from injection of 10^4^ or 10^7^ cells in the right flank, and 10^4^ cells in the left flank, were treated with intra-peritoneal anti-PD1 (100 µg per week from day 14 after allograft) or isotype control. At day 14 from allograft, they underwent surgical removal of the right tumor or a sham procedure. Tumor size was measured twice a week. Twelve animals were used in each group (6 received 10^4^ cells in the right flank, 6 received 10^7^ cells). Error bars represent the standard error for mean. (**B**) Absolute tumor volume during treatment with anti-PD1. (**C**) Tumor volume relatively to the volume at treatment initiation. (**D**) Legend for (**B**,**C**) graphes.

In order to test the synergistic effect of debulking surgery and anti-PD1 treatment, we engrafted mice with two tumor grafts: 1.10^4^ or 1.10^7^ cells in the right flank and 1.10^4^ cells in the left flank. At day 14 after engraftment, mice were randomly allocated to either a debulking surgery (group B and C) of the right tumor or a sham procedure (group A) (Fig. 2A). Mice were treated with 100 µg per week intra-peritoneal injection of isotype IgG (group B) or anti-PD1 (groups A and C). Here again, the anti-PD1 treatment effect was greater in mice receiving fewer tumor cells (Fig. 2B). When mice underwent debulking surgery and received anti-PD1 treatment (group C), the growth of the remaining left tumors was slower than the left tumors in group A (sham procedure and anti-PD1 treatment, p = 0.01). In mice that underwent the sham procedure, the growth of the left tumors was slightly faster than in mice from the corresponding debulking groups (p = 0.04).

In patients, we examined the role of metabolic tumor volume (MTV) as a surrogate marker of tumor burden and aimed to test wether MTV was associated with patients’ outcomes in second-line treatment with Nivolumab for stage IV NSCLC. Among 200 patients treated in this setting at our institution from February 2015 to July 2017, 48 had undergone 18FDG PET-scan prior to treatment. In this cohort, high MTV was associated with poor overall survival (p = 0.01) (Table 1).

**Table 1:** Metabolic tumor volume is associated with anti-PD1 treatment outcome in stage IV NSCLC patients.

	High MTV (n = 19)	Low MTV (n = 29)	p-value
**Best response**			
Partial response	4	8	
Stable disease	11	17	
Progression disease	4	4	
Progression-free survival (months)	3.1 [1.6–5.2]	5.2 [3.1–12.3]	0.13
6-months progression-free survival	3 (16%)	11 (38%)	0.049
1-year overall survival	7 (37%)	21 (73%)	**0**.**013**

**Figure 3:**
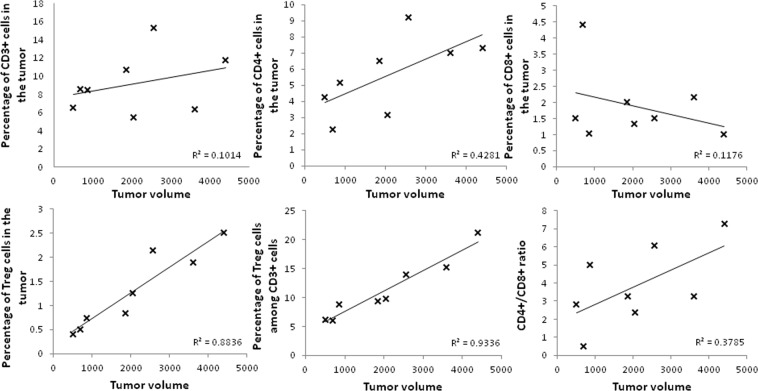
Cytometric analysis of tumors according to their volume.

We studied the immune infiltrate of KLN-205 mouse tumor allografts. Treg infiltration was positively correlated to tumor volume (Fig. 3). There was no association between tumor volume and total lymphocytes, CD8+ lymphocytes and CD4+ lymphocytes.

## Discussion

Our study showed that tumor volume is a predictive factor of anti-PD1 efficacy in lung cancer in a mouse model and based on clinical data. Moreover, partial tumor excision enhanced anti-PD1 treatment outcome in the mouse model.

Tumor volume is considered in the TNM classification of NSCLC through the T descriptor. In fact, the size of the primary tumor site has been shown to be a strong prognostic factor in NSCLC^15^. Here we used metabolic tumor volume as a surrogate marker of tumor volume in stage IV NSCLC patients and showed that it is an independent risk factor for poor survival. In melanoma, a recent report showed similar findings, with baseline tumor size being an independent prognostic factor for overall survival in patients treated with the anti-PD1 Pembrolizumab^16^.

Large tumors may lead to an immunosuppressed or tolerogenic state. We showed that bigger tumors have higher densities of T regulator lymphocytes, but similar densities of effector T cells. This may contribute to establish a tolerogenic state in large tumors. Other mechanisms of immunosuppression related to tumor volume have been reported, including metabolic competition between immune cells and cancer cells^17^ and hypoxia-induced recruitment of immunosuppressive cells and dysfunction of effector immune cells^18^.

Debulking surgery in metastatic renal cell carcinoma has long been considered a standard of care for selected patients but is not recommended in NSCLC. Nevertheless, it may be considered in the setting of immunotherapy. In fact, surgical debulking may enhance the immune system efficiency through the elevation of T-lymphocyte to tumor cell ratios, the destruction of physical barriers which prevent immune-cell infiltration of tumor, and the reduction of systemic release of immunosuppressive cytokines such as GM-CSF, IL-10, and PGE2^19^. Moreover, it has been shown that therapeutic vaccination achieves better results in small residual tumors than in larger tumors^20^. In a mouse model of mesothelioma, Broomfield *et al*. showed that partial resection of tumor enhances the effect of chemo-immunotherapy using gemcitabine and anti-CD40 antibody^21^. Tumor resection was also reported to exert a protective tumor specific CD8+ immunity in the same mesothelioma model^22^. In our study, tumor debulking achieved tumor growth delay while anti-PD1 treatment was ongoing. The effect was greater when the residual tumor was smaller. This is consistent with the abovementioned reports.

One limitation of our mouse model is that we did not take metastasis into account. In fact, tumor burden may have been underestimated since we could not perform assessment of metastasis at the time of debulking. Nevertheless, treatments were allocated randomly and post-mortem examination of control animals showed similar tumor burden between individuals (data not shown).

So far, tumor debulking in stage IV NSCLC was not considered achievable because (i) most patients have widely metastatic disease and altered PS, (ii) thoracic surgery was associated with high morbidity and (iii) onset of chemotherapy has to be delayed after surgery for recovery purpose and to lower the risk of post-operative infections, preventing efficient treatment of the non-debulked disease. Nevertheless, the development of minimally invasive techniques has minimized the morbidity and recovery time of thoracic surgery, while the good toxicity profile of immunotherapy allows its peri-operative administration. Thus, our results support the idea of tumor debulking in the context of anti-PD1 immunotherapy, in selected patients such as those with good PS and oligo-metastatic disease.

Taken together, our results suggest that tumor volume is a predictive factor of anti-PD1 efficacy in NSCLC. That effect may be mediated by T regulator lymphocytes. Tumor debulking appeared to improve treatment outcome in a mouse model and warrants further investigations in the clinical setting.

18FDG PET-scan data from patients treated with anti-PD1 for stage IV NSCLC was reviewed. MTV cut-off to segregate low *versus* high MTV patients was determined using ROC curve analysis for overall survival (AUC = 0.81, p = 0.02). Kaplan-Meier method was used to estimate progression-free survival. MTV, age, histology and Performance Status were compiled in a proportional hazard Cox model to perform multivariate analysis. Comparisons of the 6-months PFS and 1-year OS between groups were performed using Student t-test.

18FDG PET-scan data from patients treated with anti-PD1 for stage IV NSCLC was reviewed. MTV cut-off to segregate low *versus* high MTV patients was determined using ROC curve analysis for overall survival (AUC = 0.81, p = 0.02). Kaplan-Meier method was used to estimate progression-free survival. MTV, age, histology and Performance Status were compiled in a proportional hazard Cox model to perform multivariate analysis. Comparisons of the 6-months PFS and 1-year OS between groups were performed using Student t-test.

KLN-205 tumors from DBA/2 allografted mice (n = 9) were harvested at day 21 after treatment initiation. Tumors were dissociated and stained as described in the methods. Flow cytometry analysis was performed on FACS LSR Fortessa (BD biosciences, San Jose, CA, USA), and using FlowJo software (Tree Star, Ashland, OR). Cells were gated, based on forward and side scatter and on living cells. Acquisition of multiparameter data was carried out with an appropriate forward scatter (FSC) threshold to exclude debris. At least 50,000 CD3+ cells per sample were analyzed. Gating strategy is illustrated in Supplementary Fig. S1. T regulatory cells were defined as Foxp3/CD4/CD3 positive cells of all living cells.

## Supplementary information

Supplementary material

## Data Availability

The datasets used and/or analyzed during the current study are available from the corresponding author on reasonable request.
