# Supplementary material for: A rationale for surgical debulking to improve anti-PD1 therapy outcome in non small cell lung cancer
Source: Sci Rep. 2019 Nov 15;9:16902. doi: 10.1038/s41598-019-52913-z (PMC6858444; doi:10.1038/s41598-019-52913-z)
Supplement: Supplementary file 1 — Supplementary material [file 41598_2019_52913_MOESM1_ESM.doc]

A rationale for surgical debulking to improve anti-PD1 therapy outcome in non small cell lung cancer

Florian Guisier, Stephanie Cousse, Mathilde Jeanvoine, Luc Thiberville, Mathieu Salaun


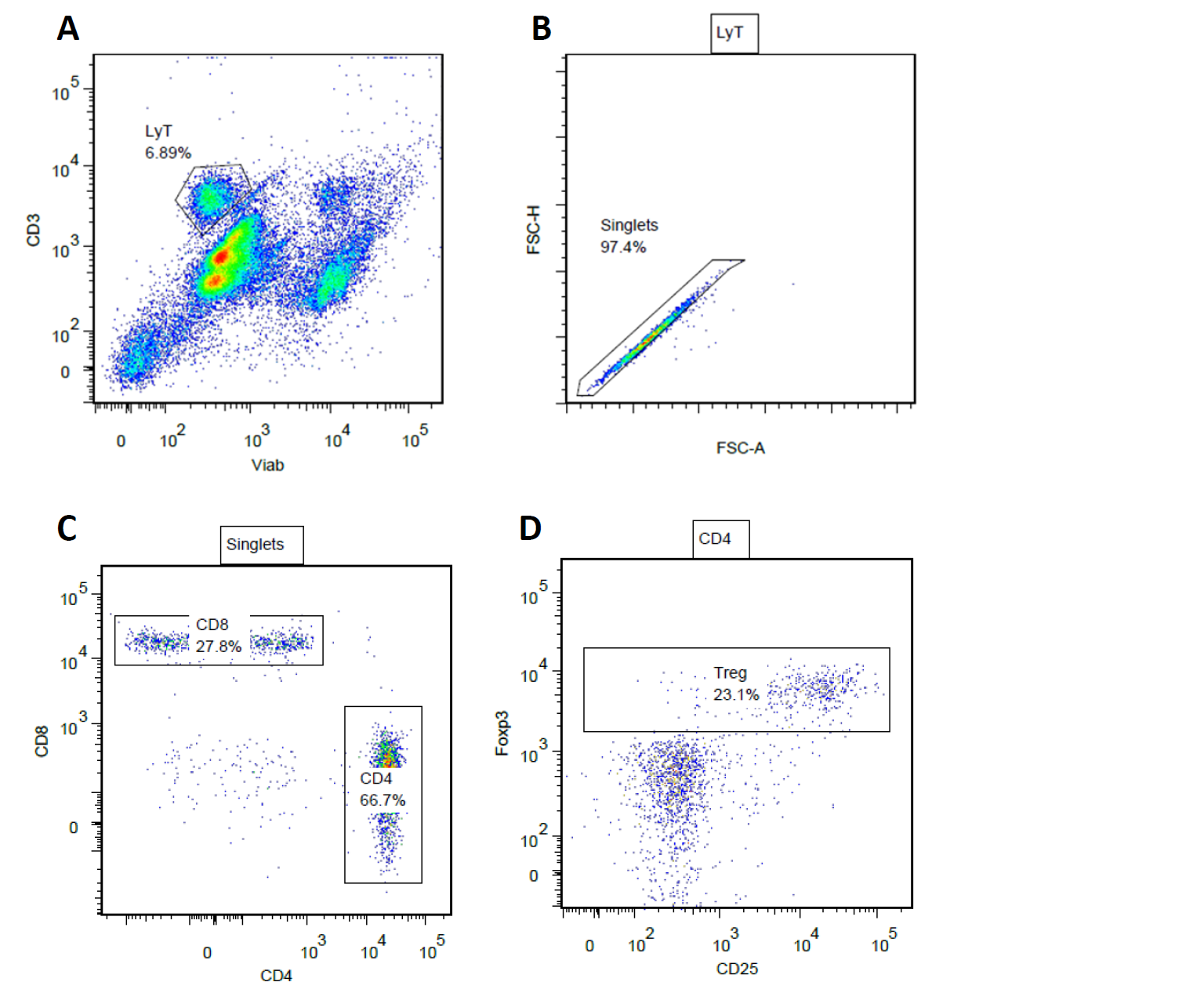


**Supplementary figure S1:** representative data plots and gating strategy for cytometric analysis of tumor infiltrating lymphocytes.

Cells (5.10^5^) were incubated with 5 μl/ml purified rat anti-mouse CD16-CD32 mAb (TruStain fcX, Biolegend Inc, San Diego, CA) before incubation with specific anti-mouse antibodies. Cells were marked with CD3-FITC, CD4-APC-Cy7, and CD25-PE (Biolegend Inc), CD8-PerCP-Cy5.5 and FoxP3-APC (eBioscience Affymetryx, Santa Clara, CA, USA), washed with PBS and fixed with 0.5% paraformaldehyde. Flow cytometry analysis was performed on FACS LSR Fortessa (BD biosciences, San Jose, CA, USA), and using FlowJo software (Tree Star, Ashland, OR). Cells were gated, based on forward and side scatter and on living cells. Acquisition of multiparameter data was carried out with an appropriate forward scatter (FSC) threshold to exclude debris. At least 50,000 CD3+ cells per sample were analyzed.

A) CD3 expression and viability dying plot to select living T lymphocytes

B) Forward scatter thresholding to exclude debris

C) CD4 and CD8 expression among T lymphocytes singlets.

D) CD25 and FoxP3 expression among CD4+ T lymphocyte singlets. T regulatory cells were defined as Foxp3/CD4/CD3 positive cells of all living cells.

Viab: viability dye. LyT: T lymphocytes. Treg: regulatory lymphocytes.

|  | Total | High MTV | Low MTV |
| --- | --- | --- | --- |
|  | (n=48) | (n=19) | (n=29) |
| Age (median) | 63.5 | 65.2 | 61.2 |
| Male | 37 (77%) | 15 (79%) | 22 (75%) |
| Smoking status |  |  |  |
| Never smoker | 5 (10%) | 1 (5%) | 4 (14%) |
| Former smoker | 26 (54%) | 14 (48%) | 12 (41%) |
| Active smoker | 17 (35%) | 4 (21%) | 13 (45%) |
| Performance status |  |  |  |
| 0-1 | 39 (81%) | 15 (52%) | 24 (83%) |
| 2 | 7 (15%) | 3 (16%) | 4 (14%) |
| Histology |  |  |  |
| Adenocarcinoma | 23 (48%) | 6 (32%) | 17 (59%) |
| Squamous cell carcinoma | 21 (44%) | 11 (38%) | 10 (34%) |
| Other | 4 (8%) | 1(3%) | 3 (10%) |
| Mutational status |  |  |  |
| KRAS | 7 (15%) | 3 (16%) | 4 (14%) |
| EGFR | 1 (2%) | 1 (3%) | 0 (0%) |
| ALK | 0 (0%) | 0 (0%) | 0 (0%) |
| Brain metastasis | 12 (25%) | 4 (21%) | 8 (28%) |

**Supplementary table S1:** characteristics of the study population at the time of anti-PD1 onset.
